# Clinical outcomes and prognostic factors in the surgical treatment of spinal dural arteriovenous fistulas: a retrospective study of 118 patients

**DOI:** 10.1038/s41598-023-45599-x

**Published:** 2023-10-25

**Authors:** Youheng Peng, Yanming Ren, Jiguang Hou, Changwei Zhang, Min He, Bowen Huang, Tengyun Chen, Jin Li

**Affiliations:** Department of Neurosurgery, West China Hospital of Sichuan University, No. 37, Guoxue Alley, Chengdu, 610041 Sichuan People’s Republic of China

**Keywords:** Risk factors, Neurological disorders, Spinal cord diseases, Neurovascular disorders

## Abstract

Spinal dural arteriovenous fistulas (SDAVFs) are the most common type of spinal vascular malformations (AVMs), constituting approximately 70% of all spinal AVMs. The impact of various clinical and radiologic features on the outcomes in patients with SDAVFs is still controversial. The purpose of the study is to investigate the clinical outcomes and prognostic factors in patients with surgically treated SDAVFs in a single center. A retrospective review was performed for all patients with SDAVFs from January 2013 to September 2021 who underwent surgery at our institution. Medical records and pre- and postoperative imaging data were analyzed. Neurological function status was evaluated by modified Aminoff-Logue Scale (mALS). Student’s t-test, Wilcoxon rank sum test, χ^2^ test and logistic regression were used to find significant prognostic factors. P values < 0.05 were considered significant. One hundred and eighteen patients were ultimately included in the study. By comparing preoperative and postoperative mALS, 69 (58.5%) patients experienced improvement, and 49 (41.5%) patients showed no improvement (worse or unchanged). Wilcoxon rank sum test showed that there was a difference between the improvement group and the no improvement group in preoperative mALS Micturition score and preoperative mALS Defecation score. The logistic regression revealed that preoperative mALS Gait score was associated with clinical improvement after surgery in patients with SDAVFs. Surgical treatment of SDAVFs is a safe and effective procedure and can lead to symptom improvement or stabilization in most patients. Preoperative neurological function status was the only factor associated with clinical prognosis.

## Introduction

Spinal dural arteriovenous fistulas (SDAVFs), also known as type I spinal arteriovenous malformations (AVMs), are rare vascular lesions with an annual incidence rate of about 5–10 cases per million people^1,2^. Despite its rarity, SDAVF is the most common type of spinal AVM, accounting for about 75–80% of all spinal AVMs^3,4^. SDAVF consists of an abnormal shunt between a dural artery branch and a radicular vein within the dura adjacent to the nerve root sheath. The arterial blood is shunted directly into the venous system, leading to chronic venous hypertension and spinal ischemia^5–7^. Once diagnosed, surgical or endovascular treatment should be performed promptly to avoid further deterioration of neurologic deficits. Although previous studies have shown that clinical and imaging characteristics, including gender, age at diagnosis, the interval from onset to treatment, fistula location, venous drainage, spinal cord edema, and preoperative neurologic severity are associated with the functional outcomes^4,8,9^, there is still no consensus on the relationship between the clinical and radiologic features and its prognosis due to their small sample sizes and lack of standardized long-term follow-up data. Therefore, it remains unclear which clinical and radiologic factors are valid indicators for clinical outcomes.

Here, we retrospectively reviewed the clinical history and prognosis of all SDAVF patients who received surgical treatment in our institution for eight years. We sought to identify the relationship between different factors and improved prognosis after surgical treatment of patients with SDAVFs.

## Methods

### Study design and setting

We reviewed all cases of SDAVFs diagnosed and treated in the West China Hospital from January 2013 to September 2021. The relevant demographic and clinical data were obtained and identified from the institutional Hospital Information System (HIS). This study was approved by the West China Hospital of Sichuan University Biomedical Research Ethics Committee. Written informed consent was obtained from each patient or the patient’s legal guardian.

### Participants, variables, and data sources

Participants met the following inclusion criteria: (1) fistula identified by spinal digital subtraction angiography (DSA); (2) available preoperative magnetic resonance imaging (MRI) of the spine; and (3) follow-up for at least one year after surgery. The exclusion criteria were as follows: (1) any prior intervention (surgical or endovascular) and (2) neurological deficits caused by other lesions.

The following data were collected: (1) demographics (age at diagnosis, gender); (2) interval from onset to treatment; (3) pre- and postoperative modified Aminoff-Logue Scale (mALS, Table 1); (4) comorbidities; (5) pre- and postoperative MR findings (intramedullary edemas, presence of an engorged venous plexus, enhancement of spinal cord parenchyma); (6) pre- and postoperative DSA findings (starting and ending segment of draining veins, a segment of fistula).

**Table 1:** Modified Aminoff and Logue’s scale.

Gait (G)	
0	Normal leg power, stance and gait
1	Leg weakness, abnormal gait, or stance but no restriction of activity
2	Restricted activity but not requiring support
3	Requiring 1 stick for walking
4	Requiring 2 sticks, crutches, or walker
5	Confined to wheelchair
Micturition (M)	
0	Normal
1	Hesitancy, urgency, frequency, altered sensation, but continent
2	Occasional urinary incontinence or retention
3	Total incontinence or persistent retention
Defecation (D)	
0	Normal
1	Mild constipation, responding well to aperients
2	Occasional incontinence or persistent constipation
3	Persistent incontinence

The length of intramedullary edemas, length of engorged venous plexus on preoperative MRI, and draining veins on DSA were calculated and represented as the number of vertebral levels spanned. Neurological function was evaluated by the mALS and identified as follows: (1) improvement: at least one of the three parts of the mALS scoring system has a decrease while the other parts have no increase in scores; and (2) no improvement: the scores of all three parts of the mALS scoring system had no change or at least one part has increased (regardless of whether the scores of other parts have decreased).

Intramedullary edemas were defined as T2 intramedullary hyperintensity; fistulas were defined as being cervical (C1-C7), upper thoracic (T1-T6), lower thoracic (T7-T12), lumbar and sacral (below T12). Patients were followed-up radiologically (MR/MRA angiography) before discharge and then at three to six months post-treatment. Patients who failed to improve after endovascular treatment underwent a follow-up DSA after a relapse was noted.

All surgical procedures were carried out by a senior neurosurgeon; clinical assessments were performed by neurologists; both radiological and clinical assessments were independently performed by two investigators, then, findings were compared, and any inconsistencies were resolved by consensus. Perioperative care and surgical procedures were all performed standardly, as reported in the literature.

The clinical follow-up was at 3, 6, and 12 months, and then once annually after surgery by the outpatient visit or phone call consultation. The mean follow-up time was 18.1 (± 6.0) months. Those patients who were lost to follow-up were excluded from the study for analysis.

### Statistical analyses

Descriptive statistics were used to analyze the frequency of participant characteristics. The mean and standard deviation was computed for normally distributed variables, and the median and interquartile range (IQR) for non-normally distributed variables. For outcome analysis, patients were grouped according to pre- to post-treatment mALS difference into (a) improvement group (mALS difference of 1 or higher); (b) no improvement group (no change or increase in mALS). Categorical variables were expressed in percentages, and χ^2^ test was used to compare the differences between ordinal and nominal variables between the two groups. Student’s t-test (2-tailed) and Wilcoxon rank sum test were used to analyze the significant difference of continuous variables between the two groups. The correlation between each factor and prognosis was assessed by the logistics regression model. Determining the optimal cut-off line of preoperative mALS score using the survminer package. All p values were two-sided. Level of statistical significance was established at < 0.05.

All statistical analyses were performed in R software version 4.2.0 (R Foundation for Statistical Computing; http://www.R-project.org, 2017).

### Ethical approval

All procedures performed were in accordance with the ethical standards of the institutional research committees and with the 1964 Helsinki Declaration and its later amendments. This study was approved by the West China Hospital of Sichuan University Biomedical Research Ethics Committee (protocol code: 1186).

### Informed consent

Written informed consent was obtained from all patients or their legal representatives to participate in the study and to publish their data.

## Results

### Clinical characteristics and outcome

**Figure 1:**
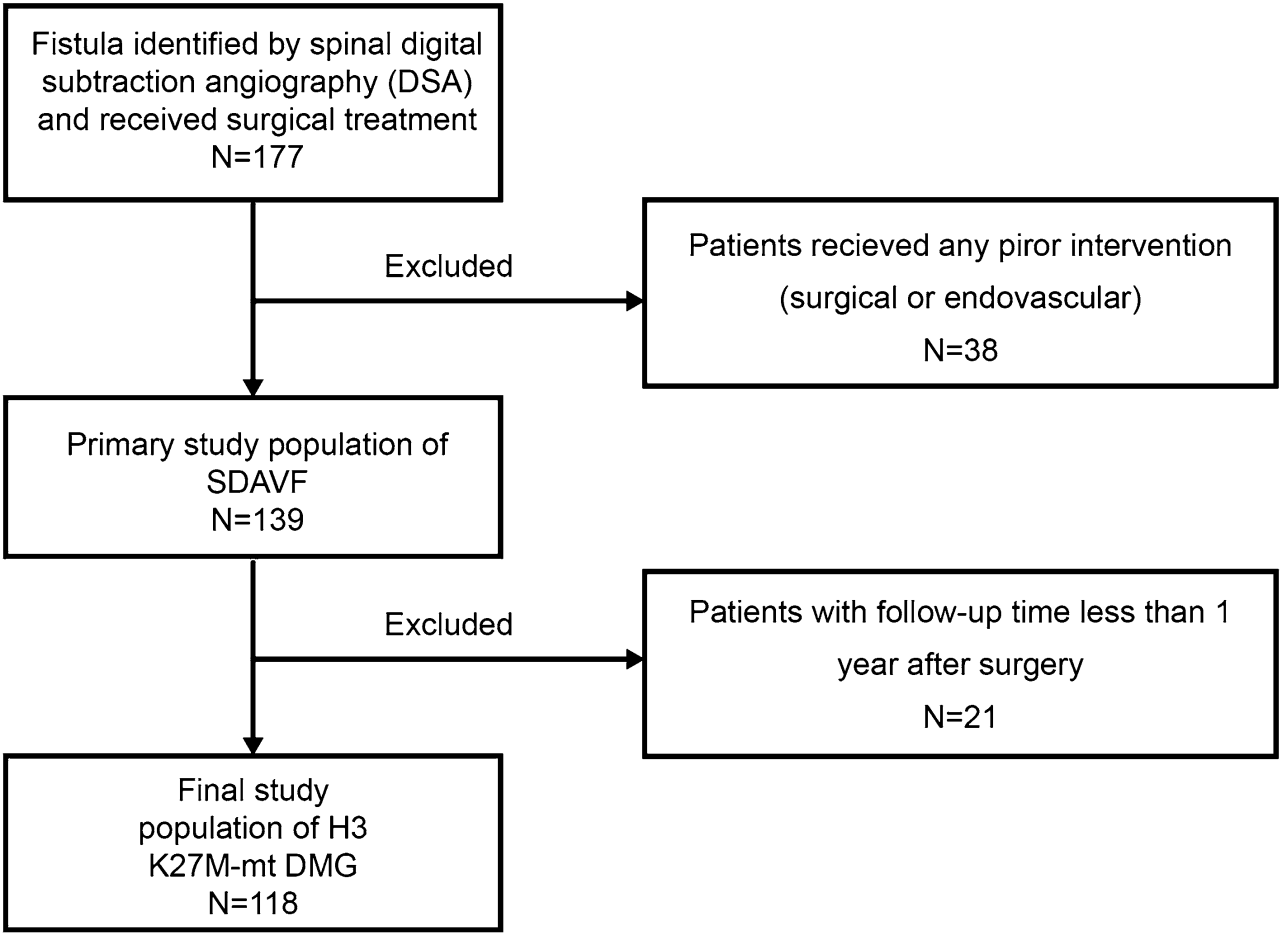
Flowchart of patient selection.

According to the exclusion criteria, 59 patients were excluded, and a total of 118 patients with SDAVFs treated surgically in our institution were included in this study ultimately. Figure 1 shows the process of patient selection for the study cohort. The baseline characteristics, including age, gender, duration of symptoms, radiologic findings, and clinical outcomes, are summarized in Table 2. The patient’s age at presentation ranged from 32 to 82 years (mean, 56.75 years), and there was a male predominance (84.4%). All patients were treated with surgery alone. The diagnosis of SDAVFs was usually delayed in most of the patients in our series, with an average symptom duration before diagnosis of 14.45 months (range 1–84 months).

**Table 2:** Baseline characteristics of SDVAFs patient cohort.

Variable	N
Gender, n (%)	
Male	104 (88.1%)
Female	14 (11.9%)
Mean age at diagnosis, years	56.75 (± 10.25)
Side, n (%)	
Left	57 (48.3%)
Right	61 (51.7%)
Interval from onset to treatment, months	14.45 (± 15.55)
Segment of fistula	
Upper thoracic (T1–T6)	14 (11.9%)
Lower thoracic (T7–T12)	65 (55.1%)
Lumbar and sacral (L1–S5)	39 (33.1%)
Length of draining veins	6.02 (± 2.95)
Length of intramedullary high signal	5.97 (± 2.60)
Length of flow voids	4.84 (± 3.02)
Enhancement of spinal cord	
Yes	67 (56.8%)
No	51 (43.2%)
Preoperative ALS gait score (0–5)	2.55 (± 1.45)
Preoperative ALS micturition score (0–3)	1.55 (± 0.93)
Preoperative ALS defecation score (0–3)	1.54 (± 0.91)
Postoperative ALS gait score (0–5)	1.97 (± 1.57)
Postoperative ALS micturition score (0–3)	1.14 (± 1.01)
Postoperative ALS defecation score (0–3)	1.13 (± 0.97)
Improvement	
Yes	69 (58.5%)
No	49 (41.5%)

The mean mALS gait score was 2.55 (± 1.45) preoperatively, with micturition and defecation scores of 1.55 (± 0.93) and 1.54 (± 0.91), respectively. After follow-up, the mean mALS gait score, micturition score, and defecation score were improved to 1.97 (± 1.57), 1.14 (± 1.01), and 1.13 (± 0.97), respectively. Wilcoxon rank sum test showed significant differences among the three scores of mALS (p < 0.0001, p < 0.0001, p < 0.0001).

### Imaging characteristic

According to preoperative spinal digital subtraction angiography (DSA), fistulas occurred most frequently at the lower thoracic spine (T7–12, n = 65, 55.1%), followed by the lumbar spine and sacral spine (below T12, n = 39, 33.1%) and the upper thoracic spine (T1–6, n = 14, 11.9%). No fistulas were located in the cervical spine. Moreover, the mean length of the draining veins was 6.02 (± 2.95).

Preoperative MRI data were available for all patients. The mean length of spinal edemas (T2-weighted image hyperintensity) was 5.97 (± 2.60), and enhanced vessel mean length on preoperative T1-weighted image was 4.84 (± 3.02).

### Risk factors of patients with SDAVF

According to the pre- and postoperative mALS, patients were divided into two subgroups: improvement and no improvement. At the last follow-up, 69 patients (58.5%) experienced improvement, and 49 patients (41.5%) showed no improvement (19 people (16.1%) got worse, and 30 people (25.4%) remained unchanged). Table 3 showed the baseline characteristics of the two groups. χ^2^ test, t-student test, and Wilcoxon rank sum test showed significant differences among the two groups on preoperative mALS micturition score (p < 0.0001) and preoperative mALS defecation score (p < 0.0001). It should be noted that the preoperative mean mALS score of the improved group (gait 2.59 ± 1.40, micturition 1.74 ± 0.90, defecation 1.74 ± 0.89) is higher than that of the no improved group (gait 2.49 ± 1.54, micturition 1.29 ± 0.91, defecation 1.27 ± 0.88), and we will discuss this in the discussion part. No significant differences were found for other clinical or radiologic variables.

**Table 3:** Baseline characteristics of SDAVFs patients in the improvement group and no improvement group.

Variable	Improvement n = 69	No Improvement n = 49	p Value
Gender, n (%)			
Male	60 (87.0%)	44 (89.8%)	0.8562**
Female	9 (13.0%)	5 (10.2%)	
Mean age at diagnosis, years	56.39 (± 9.38)	57.24 (± 11.45)	0.6687*
Side, n (%)			
Left	29 (42.0%)	28 (57.1%)	0.1521**
Right	40 (58.0%)	21 (42.9%)	
Interval from onset to treatment, months	13.41 (± 13.42)	15.10 (± 15.65)	0.5400*
Segment of fistula			
Upper thoracic (T1–T6)	8 (11.6%)	6 (12.2%)	0.1081**
Lower thoracic (T7–T12)	33 (47.8%)	32 (65.3%)	
Lumbar and sacral (L1–S5)	28 (40.6%)	11 (22.4%)	
Length of draining veins	5.65 (± 2.75)	6.53 (± 3.17)	0.0969***
Length of intramedullary high signal	6.14 (± 2.61)	5.71 (± 2.59)	0.5270***
Length of flow voids	4.57 (± 2.97)	5.22 (± 3.08)	0.1697***
Enhancement of spinal cord			
Yes	42 (60.9%)	25 (51.0%)	0.3812**
No	27 (39.1%)	24 (49.0%)	
Preoperative ALS gait (0–5)	2.59 (± 1.40)	2.49 (± 1.54)	0.6351***
Preoperative ALS micturition (0–3)	1.74 (± 0.90)	1.29 (± 0.91)	0.0061***
Preoperative ALS defecation (0–3)	1.74 (± 0.89)	1.27 (± 0.88)	0.0034***
Postoperative ALS gait (0–5)	1.39 (± 1.26)	2.80 (± 1.61)	< 0.0001***
Postoperative ALS micturition (0–3)	0.77 (± 0.75)	1.67 (± 1.09)	< 0.0001***
Postoperative ALS defecation (0–3)	0.75 (± 0.74)	1.65 (± 1.03)	< 0.0001***
Follow up time (months)	19.76 (± 5.78)	15.84 (± 5.65)	0.0005***

Significant values are in bold.

*p value calculated using the t-test.

**p value calculated using the chi-square test.

***p value calculated using the Wilcoxon rank-sum test.

Multivariate logistic regression analysis was conducted to further evaluate whether there is any relationship between clinical imaging characteristics and clinical outcomes. The results showed that lower preoperative mALS gait score was associated with functional improvement (p = 0.0406, odds ratio, 1.4867; 95% confidence interval, 1.0291–2.2146). Table 4 showed the results of the multivariate logistic regression analysis. The optimal cut off line for preoperative mALS score calculated through the survminer package is 4.5 (sensitivity 0.721, specificity 0.560), which means that patients with a preoperative mALS score of five or more may have worse prognosis.

**Table 4:** Logistic regression analysis for patient cohort.

Predictor	OR	95% CI	p value
Side (right vs. left)	0.5199	0.2099–1.2525	0.1486
Gender (female vs. male)	0.5569	0.1169–2.4003	0.4410
Age at diagnosis	1.0100	0.9641–1.0589	0.6740
Interval from onset to diagnosis (month)	1.0006	0.9660–1.0369	0.9725
Segment of fistula			
Lower thoracic vs. upper thoracic	1.8006	0.4870–7.0838	0.3828
Lumbar and sacral vs. upper thoracic	0.2814	0.0560–1.3316	0.1123
Length of draining veins	1.1727	0.9878–1.4071	0.0747
Length of intramedullary high signal	0.8747	0.7153–1.0598	0.1784
Length of flow voids	1.1666	0.9805–1.3961	0.0825
Enhancement of spinal cord parenchyma (no vs. yes)	1.9733	0.7864–5.0737	0.1499
ALS gait (0–5)	1.4867	1.0291–2.2146	**0.0406**
ALS micturition (0–3)	1.4044	0.1157–32.4733	0.7925
ALS defecation (0–3)	0.2884	0.0117–3.8140	0.3542

Significant values are in bold.

Interestingly, the Wilcoxon rank sum test showed that the mALS gait score showed no significant difference between the two groups, but it was shown to be associated with poor prognosis in logistic regression model, while this situation is reversed in mALS micturition and defecation score.

## Discussion

Surgical treatment of SDAVF is related to complete cure in the vast majority of patients^10,11^. As a minimally invasive treatment, endovascular therapy can usually be combined with diagnostic procedures. In the past few years, intravascular techniques and materials have been generally improved, and new liquid embolic agents, such as Onyx, have shown good results in the transarterial embolization of cranial DAVFs. On the other hand, the surgical technique has also been improved, with better preoperative fistula localization, and the new minimally invasive techniques have been applied. In 2004, Steinmetz et al. published a meta-analysis that reported that the success rate of the endovascular treatment group was 46%^12^, while in a series of recent studies, the observed success rate was more than 80%^13^, reflecting advances in intravascular technology, but even so, the first failure rate and late recurrence rate of endovascular therapy were still higher than those of surgery^7,12,13^. The failure of endovascular treatment is mainly due to the fact that the glue does not reach the draining vein^14^. In practice, it could be observed that it may be more difficult to reach the draining intradural vein through intravascular approach. In some cases with complex fistula architecture, endovascular therapy may be difficult to operate in microvessels, and the difficulty in controlling the embolization range of glue leading to poor fistula closure is also the possible reason for its low success rate^15,16^.

Divide the evaluation of neurological function into two groups: improvement and no improvement, rather than dividing deterioration into the third groups independently. Because for patients, on the premise of bearing the cost and time of surgery and postoperative rehabilitation, whether the postoperative neurological function is not improved or deteriorated, the surgery will be considered unsuccessful by the patient. Based on similar reasons, once one of the three neurological functions, gait, micturition, and defecation, deteriorated, regardless of whether other neurological functions recovered, it will affect the patient's quality of life, leading to patients believing that surgery is not entirely successful.

Previous research on prognostic factors has been limited by their scale. To our knowledge, this study is the largest retrospective study aiming to provide prognostic factors of patients with SDAVFs in a single institution. Our data showed that about one half (57/118, 48.3%) of our patients had improvement in motor function after treatment. However, the proportion of patients who recovered urination (50/118, 42.4%) and defecation (49/118, 41.5%) function after surgery is lower. These findings agree with a 2004 meta-analysis of patients with SDAVFs^12^.

Consistent with previous studies^4,17,18^, our study showed that SDAVFs was more common in men (104/118, 88.1%), and the mean age of our patient cohort is 56.75 (± 10.25) years old, but no association was found between sex or age at diagnosis with clinical improvement. The duration of symptoms is also considered to be a controversial factor^9,19^. Some studies believed that prolonged duration of symptoms will have adverse effects on the prognosis. However, the negative results in both the χ^2^ test (p = 0.5400) and logistic regression model (p = 0.9725) in our study did not support the prognostic value of symptom duration.

Whether preoperative imaging results can be used as a prognostic factor of SDAVF was also controversial^20–22^. MRI results were pathological in all of our patients. Similar to most other studies^21–23^, in our patients’ cohort, fistulas were most common in the lower thoracic segment (T7–T12). Gilbertson and Cenzato et al. believed that SDAVFs was more sensitive to treatment when the fistula located in the lower thoracic segment of the spinal cord^8,23^. However, in our study, whether in χ^2^ test (p = 0.1081) or logistic regression analysis (vs. upper thoracic, p = 0.3828), the segment of the fistula had no significant impact on the prognosis. The same conclusion also applied to the length of drainage veins displayed (p = 0.0747) on DSA and length of intramedullary high signal (p = 0.1784), length of flow voids (p = 0.0825), and enhancement of spinal cord parenchyma (p = 0.1499) on MRI. In addition, we also calculated Pearson correlation coefficients between mALS and various clinical and imaging factors to verify their correlation (shown in Supplementary Table S1). The result showed that only the extent of spinal edema was correlated with the ALS gait score (p = 0.01).

There is no doubt that the postoperative mALS in the no improved group is higher than that in the improvement group (p < 0.0001), but the preoperative mALS, especially the micturition and defecation score in the no improved group, is significantly lower than that in the improved group, which seems to be contrary to the conclusion in previous studies that poor preoperative neurological function leads to poor prognosis^8,17,24,25^.

We believe that this may be due to those patients with poor preoperative neurological status are more difficulty to further deteriorate after surgery and patients with more severe preoperative functional impairment had higher chances for improvement, so it finally showed that patients with higher mALS have better functional improvement than patients with lower mALS, which is similar to the conclusion of another prospective study^18^. Logistic regression analysis can further study the relationship between the variables and more objectively reflect the impact of the variables on the prognosis. Therefore, we conducted a logistic regression analysis to further explore the impact of preoperative neurological function and other clinical and imaging factors on the prognosis. The final results are also similar to previous studies: preoperative neurological function, especially motor function, is related to prognosis, and its deterioration is often related to poor prognosis (OR = 1.4867, p = 0.0406)^4,17^. Apart from it, logistic regression analysis did not show that other variables were related to prognosis.

## Strengths and limitations

As a retrospective study, a risk of selection bias was present, and our patient cohort might not be truly representative of all patients with SDAVFs. Second, the follow-up time of our study was one year, and the risk factors for the long-term prognosis of SDAVFs patients have not been identified. In the future, a longer follow-up should be conducted for further research.

## Conclusion

SDAVF is the most common spinal vascular malformation. Surgical treatment is a safe and effective treatment for SDAVF, which has been verified. This study reviewed and analyzed the clinical and imaging data from a large number of patients with SDAVFs in a single center, and the result showed that preoperative neurological function status was the only factor associated with clinical prognosis. However, the clinical and imaging results, such as age, sex, lesion side, fistula segment, spinal cord edema, and length of drainage vein, seemed to be unrelated to the prognosis.

### Supplementary Information

Supplementary Table 1.

## Data Availability

The datasets generated during and/or analyzed during the current study can be obtained from the corresponding author (Jin Li) 3-years after date-of-publication.
