# Supplementary material for: Clinical outcomes and prognostic factors in the surgical treatment of spinal dural arteriovenous fistulas: a retrospective study of 118 patients
Source: Sci Rep. 2023 Oct 25;13:18266. doi: 10.1038/s41598-023-45599-x (PMC10600191; doi:10.1038/s41598-023-45599-x)
Supplement: Supplementary file 1 — Supplementary Table 1. [file 41598_2023_45599_MOESM1_ESM.docx]

Supplementary Table 1: Pearson correlation coefficients and p values between mALS and various clinical and imaging factors

| **Variable** | Preoperative ALS Gait Score | | Preoperative ALS Micturition Score | | Preoperative ALS Defecation Score | |
| --- | --- | --- | --- | --- | --- | --- |
|  | correlation coefficient | p value | correlation coefficient | p value | correlation coefficient | p value |
| **Side** | -0.03 | 1.0 | 0.03 | 1.0 | 0.05 | 1.0 |
| **Gender** | 0.08 | 1.0 | -0.05 | 1.0 | -0.05 | 1.0 |
| **Age at diagnosis** | 0.00 | 1.0 | -0.05 | 1.0 | -0.07 | 1.0 |
| **Interval from onset to diagnosis** | 0.03 | 1.0 | 0.03 | 1.0 | 0.04 | 1.0 |
| **Segment of fistula** | 0.09 | 1.0 | 0.12 | 1.0 | 0.12 | 1.0 |
| **Length of draining veins** | -0.03 | 1.0 | 0.01 | 1.0 | 0.00 | 1.0 |
| **Length of intramedullary high signal** | 0.35 | **0.01** | 0.26 | 0.32 | 0.25 | 0.43 |
| **Length of flow voids** | -0.16 | 1.0 | -0.09 | 1.0 | -0.09 | 1.0 |
| **enhancement of spinal cord parenchyma** | 0.15 | 1.0 | 0.07 | 1.0 | 0.06 | 1.0 |
